# Supplementary material for: The long noncoding RNA lncCIRBIL disrupts the nuclear translocation of Bclaf1 alleviating cardiac ischemia–reperfusion injury
Source: Nat Commun. 2021 Jan 22;12:522. doi: 10.1038/s41467-020-20844-3 (PMC7822959; doi:10.1038/s41467-020-20844-3)
Supplement: Supplementary file 1 — Supplementary Information [file 41467_2020_20844_MOESM1_ESM.pdf]

Supplementary materials

The long non-coding RNA lncCIRBIL disrupts the nuclear translocation of Bclaf1 alleviating cardiac ischemia/reperfusion injury

Short title: LncCIRBIL regulates cardiac I/R injury via Bclaf1

Yang Zhang[1,#], Xiaofang Zhang[1,#], Benzhi Cai[1,#], Ying Li[1], Yuan Jiang[1], Xiaoyu Fu[1], Yue Zhao[1], Haiyu Gao [1], Ying Yang [1], Jiming Yang[1], Shangxuan Li[1], Hao Wu[1], Xuexin Jin[1], Genlong Xue[1], Jiqin Yang[1], Wenbo Ma[1], Qilong Han[1], Tao Tian[1], Yue Li[2, 3], Baofeng Yang[1,4*], Yanjie Lu[1], Zhenwei Pan [1, 4*]

1. Department of Pharmacology (The Key Laboratory of Cardiovascular Research, Ministry of Education) at College of Pharmacy, Harbin Medical University, Harbin 150086, China.

2. Institute of Metabolic Disease, Heilongjiang Academy of Medical Science, Harbin Medical University Cancer Hospital, Harbin, China.

3. Department of Cardiology, The First Affiliated Hospital, Harbin Medical University, Youzheng Street 23#, Nangang District, Harbin, 150001, Heilongjiang Province, People's Republic of China.

4. Research Unit of Noninfectious Chronic Diseases in Frigid Zone, Chinese Academy of Medical Sciences, 2019RU070

* Corresponding author: Prof. Zhenwei Pan. panzw@ems.hrbmu.edu.cn. or Prof. Baofeng Yang. yangbf@ems.hrbmu.edu.cn. Department of Pharmacology, College of

Pharmacy, Harbin Medical University, Baojian Road 157, Harbin, Heilongjiang

150081, P. R. China

Fax: 86 451 86675769 or 86667511

Tel: 86 451 86671354

# With equal contributions to the work

Supplementary Figure 1 The genomic location and sequence of lncRNA NONMMUT058343

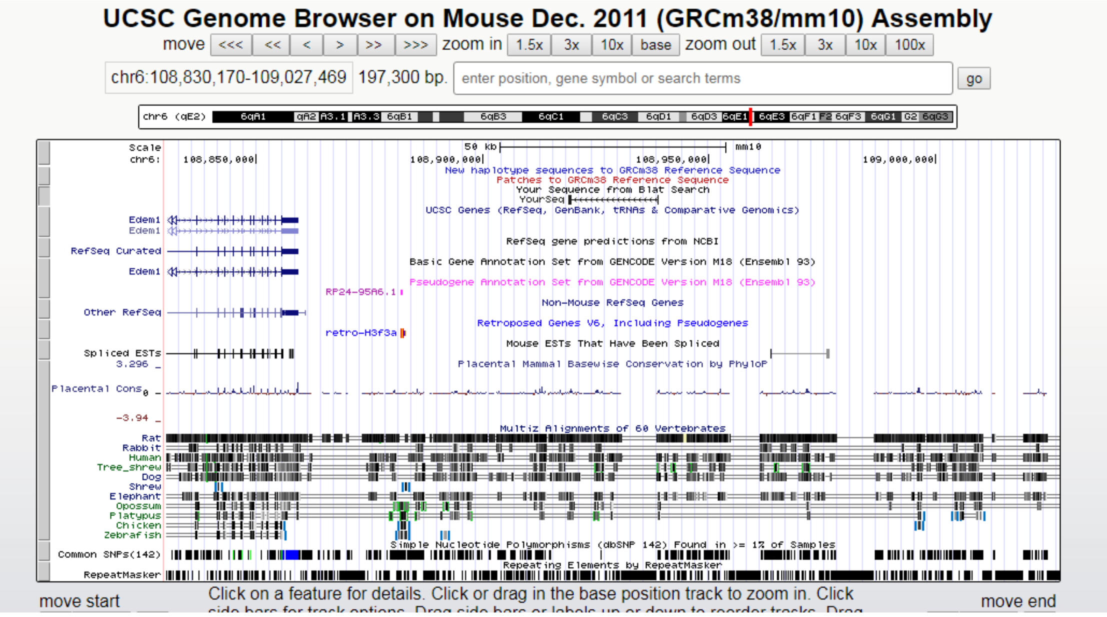

Sequence of NONMMUT058343.2 Chr6(108918953~108938686) 862nts

TGCTTAACCAGTGAGCATAGAAATGGACGTGCTCTGAGTAAGTGTTTAACA

GTCCTCTATCTAAGCTGCTTCTTCTGTGGCATGCATTACACAGGCACTGTAC

TGAATGTCAGAGAGCAGGCATTTCGAACTGGAATTTCATGGTGTAAAAATA

AAGGCCTTGTTATTGACTCTGAGAACCATGGGAACAAATTAAACACAGCTT

AATAGCACAAAGGGGAATGATGGGGACATTCGGAGCCTAATAAGGTGATCC

ATGGGGTTCATTAGCACAGGCAGAGCTTATCAGAAGAGGCCTCAGAACCTA

ATTAGCTGAACCCCCATCAAATAGATGCATTTGATTAATATGACTCCATTCTG

AGTTTAAACATCAACATTAATTAGCCCCATAATTGATAGATTTCCCAGCCATG

TGAGGAACATTCATCATACTACTCAAGAAAGATCTTTTTTTTTCCTGATATT

TCAAGACAGGTTCTCTCTGGGTAGTCCAGACTGACCTCAAACTAACACTCT

TCCAGCCTTGCTTTCAGGGATGCTGGGATTATAAGTATGTGCCACCATCTAC

AACAAGATCTGTCTTAATATGTAAAGCCTTTGAAAACTGAAAACCTTAGGC

CAATGAGACAGCTCCCTGGGCTCTTGGCATGGAAATCTGAAATCCTTAGAA

GGAGAGAACTAACTCCCCAAAACTGTCCTCTGACATCCCATCTACACACAC

ACACACACACACACACACACACACACACACAATGAACAAGAAAAAGAAG

AAGAAGGAGGAGGACGAGGAGGAGGACGAGGAGGATGAGGACAAGGAG

GAAGAAGAAGAAGAAAGAAAGAAAGAAAGAAAGAAAGAAAGAA

Supplementary Figure 2. The conservative sequence of LncCIRBIL in human genome

A. The sequence in red is the longest one obtained when blasted LncCIRBIL sequence with human genome

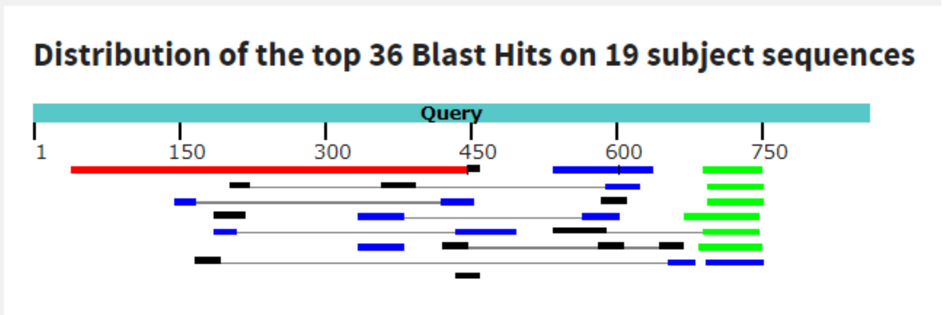

B. Complementarity between human and mouse lncCIRBIL.

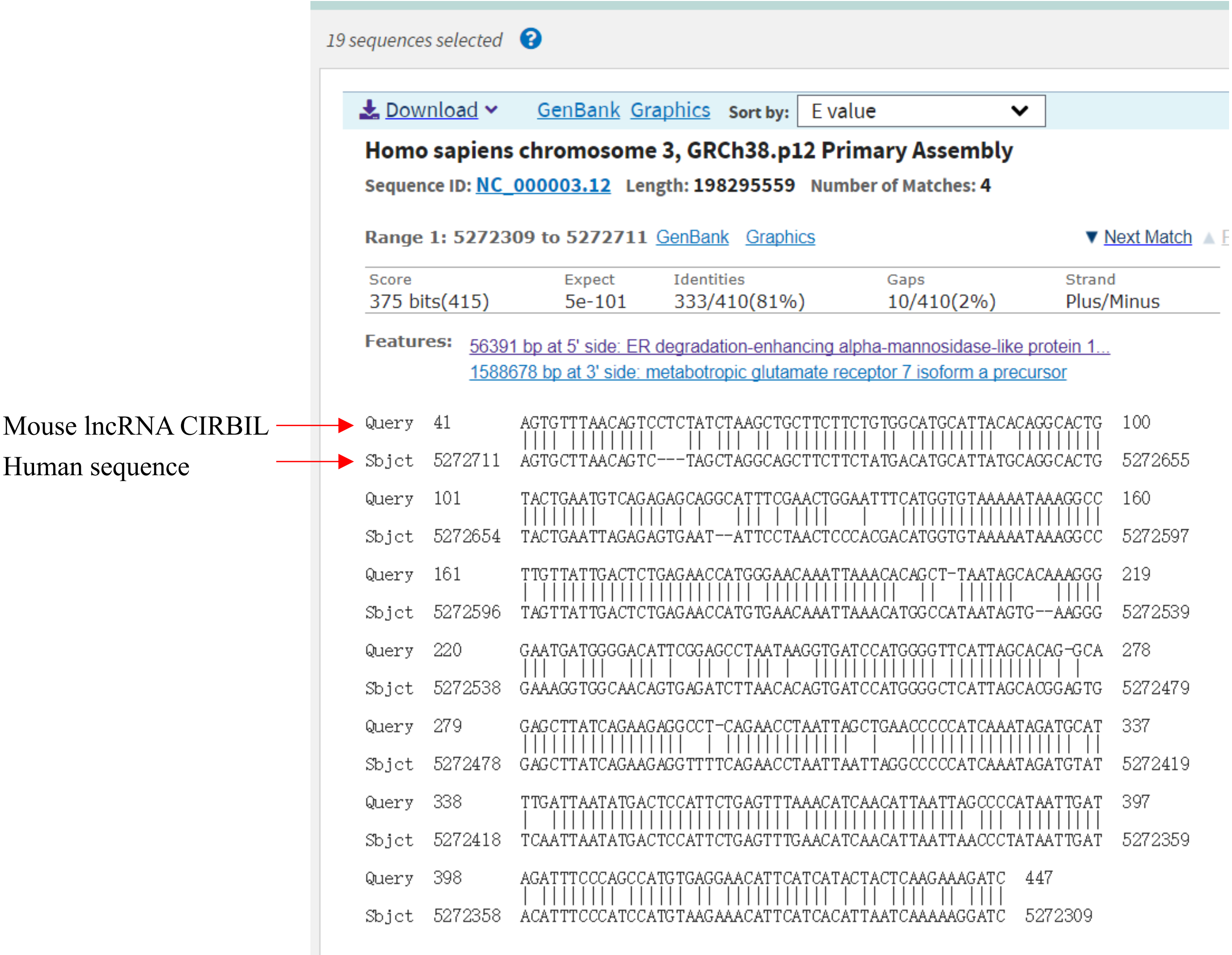

C. Conservative sequence of human CIRBIL

AGTGCTTAACAGTCTAGCTAGGCAGCTTCTTCTATGACATGCATTATGCAGG
CACTGTACTGAATTAGAGAGTGAATATTCCTAACTCCCACGACATGGTGTAA
AAATAAAGGCCTAGTTATTGACTCTGAGAACCATGTGAACAAATTAAACAT
GGCCATAATAGTGAAGGGGAAAGGTGGCAACAGTGAGATCTTAACACAGT
GATCCATGGGGCTCATTAGCACGGAGTGGAGCTTATCAGAAGAGGTTTTCA
GAACCTAATTAATTAGGCCCCCATCAAATAGATGTATTCAATTAATATGACTC

CATTCTGAGTTTGAACATCAACATTAATTAACCCTATAATTGATACATTTCCC
ATCCATGTAAGAAACATTCATCACATTAATCAAAAAGGATC
407 nts

Supplementary Figure 3

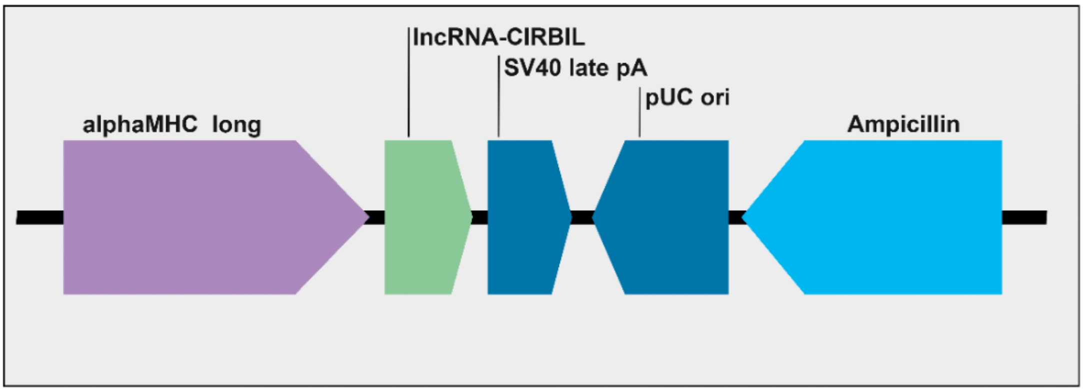

Supplementary Figure 3. The cardiac-specific lncCIRBIL Tg mice. The sequence of lncCIRBIL was cloned into the murine α-MHC promoter expression vector and the obtained DNA fragment containing lncCIRBIL driven by α-MHC promoter was microinjected into fertilized eggs.

Supplementary Figure 4

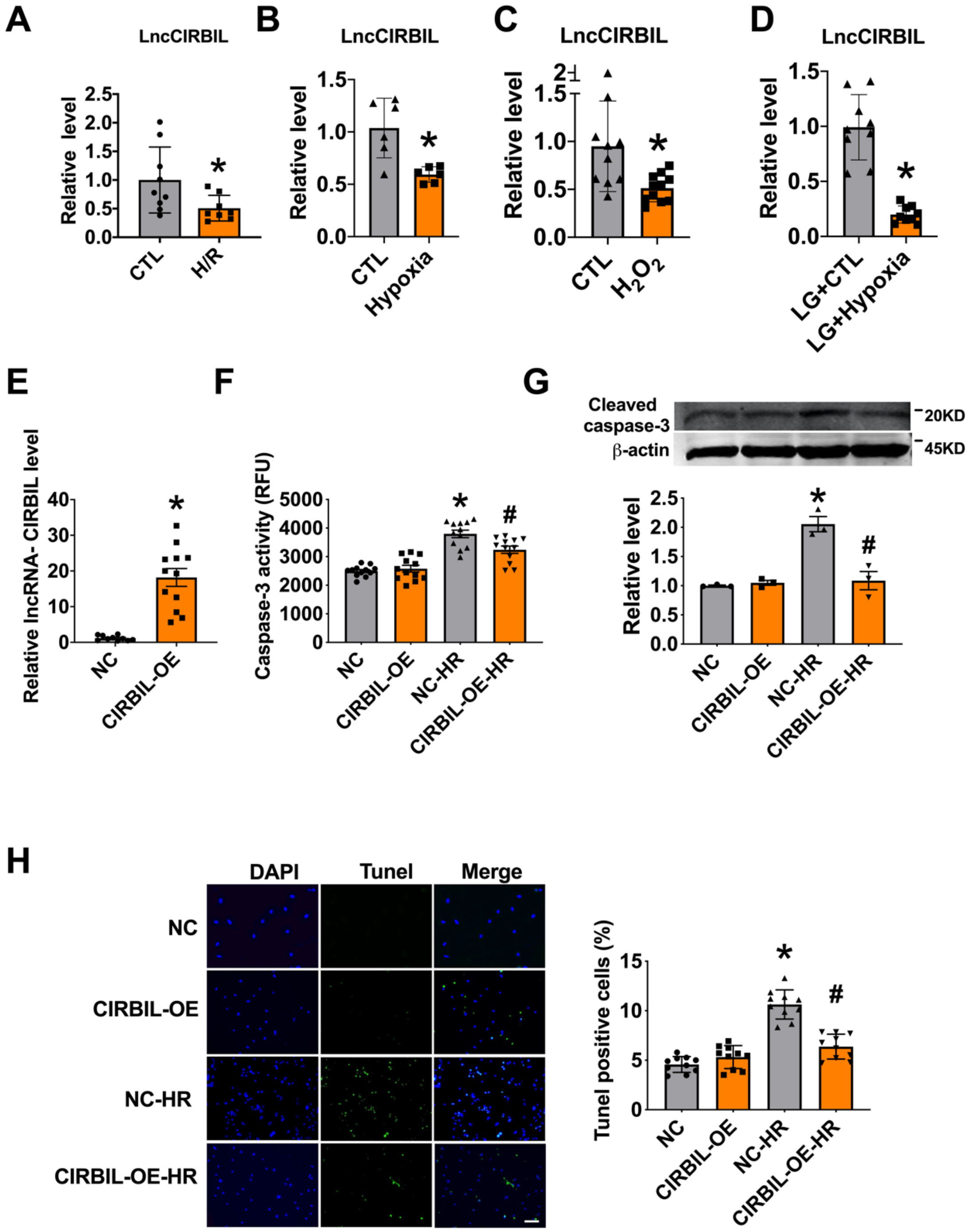

Supplementary Figure 4. Overexpression of LncCIRBIL mitigated hypoxia/reoxygenation (H/R) induced injury of cultured cardiomyocytes. (A) The levels of lncCIRBIL in cardiomyocytes exposed to H/R. CTL N=9, H/R N=8 from 3 independent cultures. *P<0.05 versus control (CTL). P-values were determined by unpaired t test. **(B)** The levels of lncCIRBIL in cardiomyocytes exposed hypoxia. N=6 from 3 independent cultures. *P<0.05 versus control (CTL). P-values were

determined by unpaired t test. **(C)** The levels of lncCIRBIL in cardiomyocytes exposed to hydrogen peroxide (H_2O_2). CTL N=10, H_2O_2 N=11 from 3 independent cultures. **P*<0.05 versus control (CTL). P-values were determined by unpaired t test. **(D)** The levels of lncCIRBIL in cardiomyocytes exposed to hypoxia with energy deprivation (low glucose). N=9 from 3 independent cultures. **P*<0.05 versus control (CTL). P-values were determined by unpaired t test. **(E)** Verification of the expression of lncCIRBIL in the cardiomyocytes after transfection of lncCIRBIL plasmid. NC N=10, CIRBIL-OE N=12 from 3 independent cultures. **P*<0.05 versus NC (negative control, empty plasmid). P-values were determined by unpaired t test. **(F, G)** Effects of lncCIRBIL on caspase-3 activity and cleaved caspase-3 protein level. N=12 from 3 independent cultures for caspase-3 activity, and N=3 for cleaved caspase-3 protein level. **P*<0.05 versus NC; $^{\#}P$<0.05 versus NC-HR. P-values were determined by one-way ANOVA test followed by Bonferroni post hoc analysis. **(H)** LncCIRBIL overexpression reduced the H/R-induced apoptosis by TUNEL staining (scale bar: 20μm). N=10 from 3 independent cultures. *P<0.05 versus NC; $^{\#}P$<0.05 versus NC-HR. P-values were determined by one-way ANOVA test followed by Bonferroni post hoc analysis. Data represent the mean ± SEM, n numbers are given in parentheses.

Supplementary Figure 5

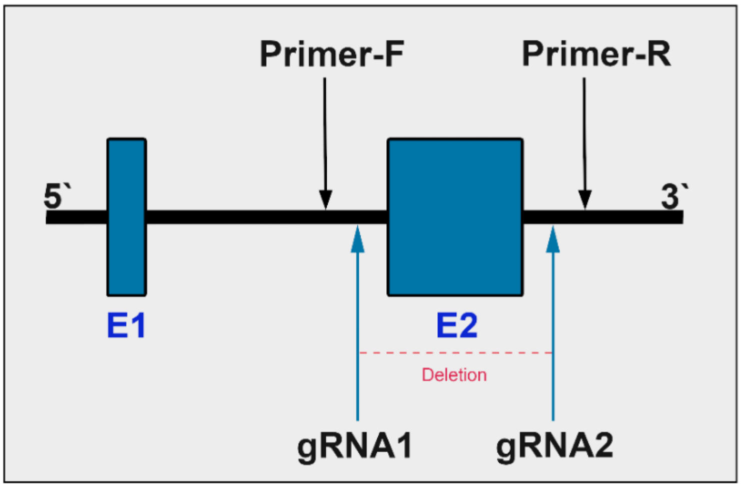

Supplementary Figure 5. The lncCIRBIL knockout mice. The chr6_108868947-108888680 gene is located on mouse chromosome 6. Two exons have been identified. Exon 2 was selected as target site (sequences shown on the next page). Cas9 mRNA and gRNA generated by in vitro transcription were then injected into fertilized eggs for KO mouse productions. The founders were genotyped by PCR followed by DNA sequencing analysis. The positive founders were breeding to the next generation which was genotyped by PCR and DNA sequencing analysis.

Supplementary Figure 6

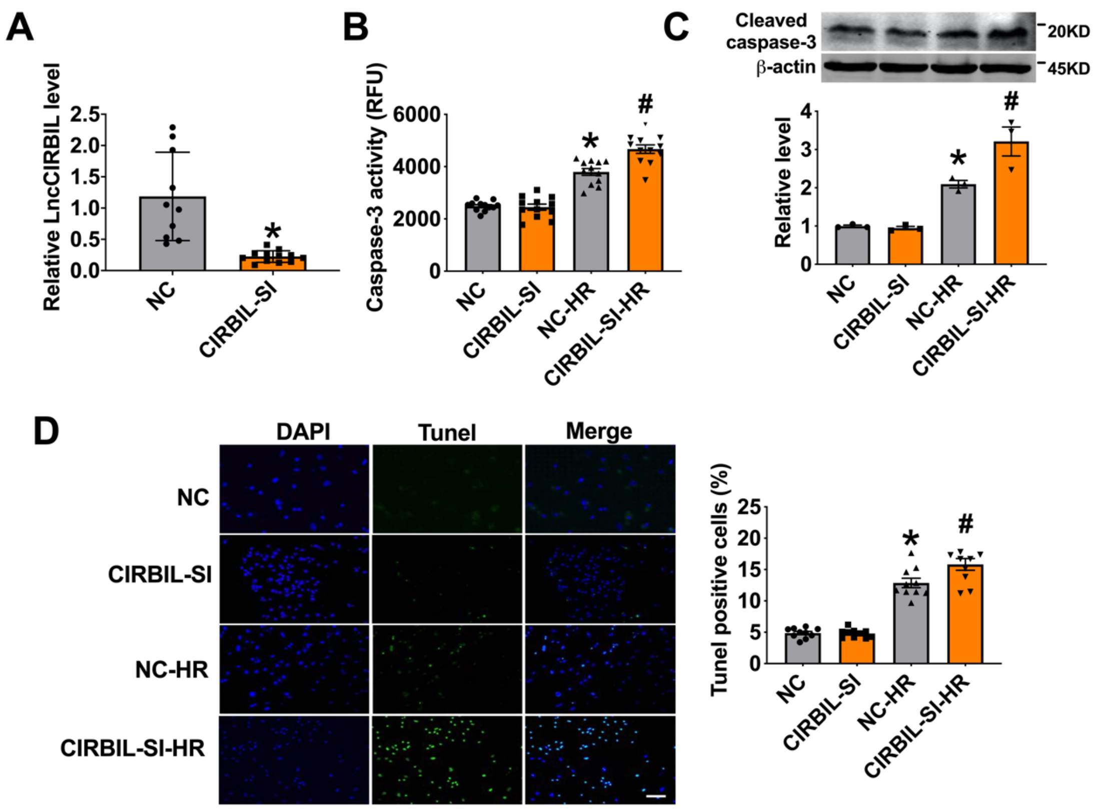

Supplementary Figure 6. Downregulation of LncCIRBIL exacerbated hypoxia/reoxygenation induced cardiac myocyte injury. (A) LncCIRBIL in the cardiomyocytes after transfection lncCIRBIL siRNA. NC N=10, CIRBIL-SI N=12 from 3 independent cultures. **P*<0.05 versus NC group. P-values were determined by unpaired t test. **(B, C)** Caspase-3 activity and cleaved caspase-3 protein level. N=12 from 3 independent cultures for caspase-3 activity, and N=3 for cleaved caspase-3 protein level. **P*<0.05 versus NC; #*P*<0.05 versus NC-HR. P-values were determined by one-way ANOVA test followed by Bonferroni post hoc analysis. **(D)** Apoptosis evaluated by TUNEL staining (scale bar: 20μm). N=10 from 3 independent cultures. **P*<0.05 versus NC; #*P*<0.05 versus NC-HR. P-values were determined by one-way ANOVA test followed by Bonferroni post hoc analysis. Data represent the mean ± SEM, n numbers are given in parentheses.

Supplementary Figure 7

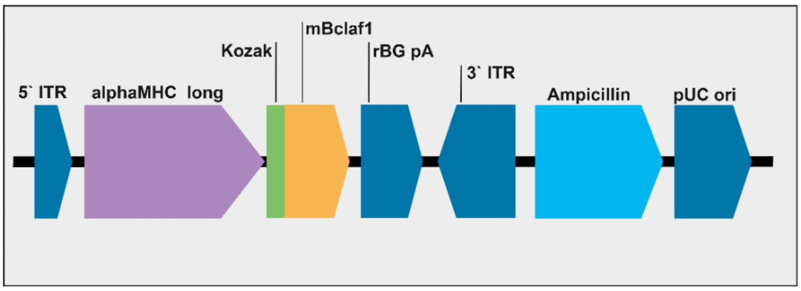

Supplementary Figure 7. The cardiac-specific Bclaf1 transgenic mice. A transgene containing Bclaf1 was cloned downstream of the a-MHC promoter. A fragment containing the promoter and transgene was agarose gel-purified and used in a microinjection of the pronucleus of one-cell mouse embryos of C57BL/6 mice.

Supplementary Figure 8

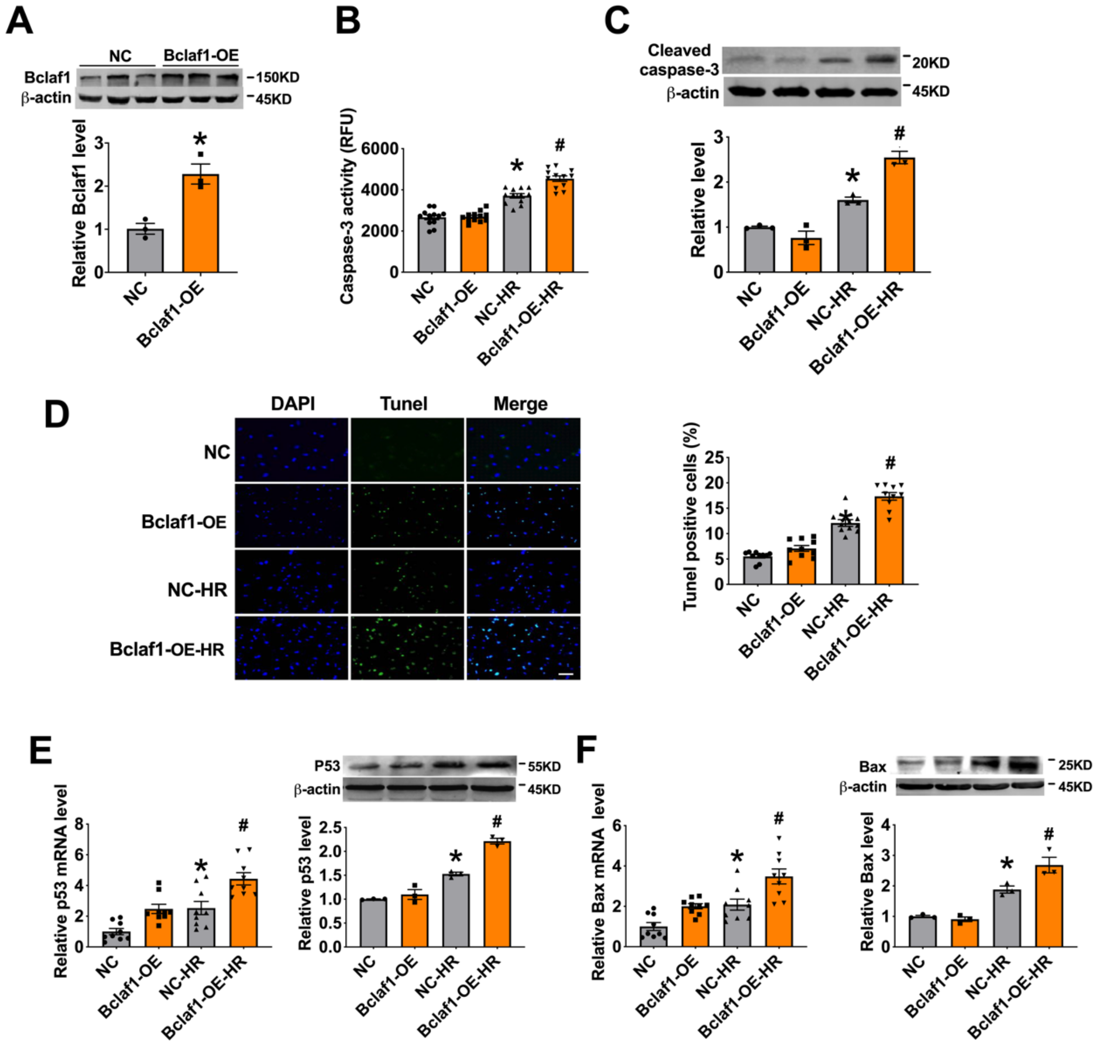

Supplementary Figure 8. Overexpression of Bclaf1 promoted apoptosis of cardiomyocytes subjected to hypoxia/reoxygenation. (A) Bclaf1 protein level in cardiomyocytes transfected with Bclaf1 overexpressing plasmids. N=3. *P<0.05 versus NC (negative control, empty plasmid). P-values were determined by unpaired t test. **(B, C)** Caspase-3 activity and cleaved caspase-3 protein level. N=12 from 3 independent cultures for caspase-3 activity, and N=3 for cleaved caspase-3 protein level. *P<0.05 versus NC, $^{\#}P$<0.05 versus NC-HR. P-values were determined by one-way ANOVA test followed by Bonferroni post hoc analysis. **(D)** Cardiomyocyte apoptosis by TUNEL staining (scale bar: 20μm). N=10 form 3 independent cultures. *P<0.05 versus NC, $^{\#}P$<0.05 versus NC-HR. P-values were determined by one-way ANOVA test followed by Bonferroni post hoc analysis. **(E, F)** The mRNA and

protein levels of p53 and Bax. N=9 for PCR and N=3 for Western blot, from 3 independent cultures. **P*<0.05 versus NC, #*P*<0.05 versus NC-HR. P-values were determined by one-way ANOVA test followed by Bonferroni post hoc analysis. Data represent the mean ± SEM, n numbers are given in parentheses.

Supplementary Figure 9

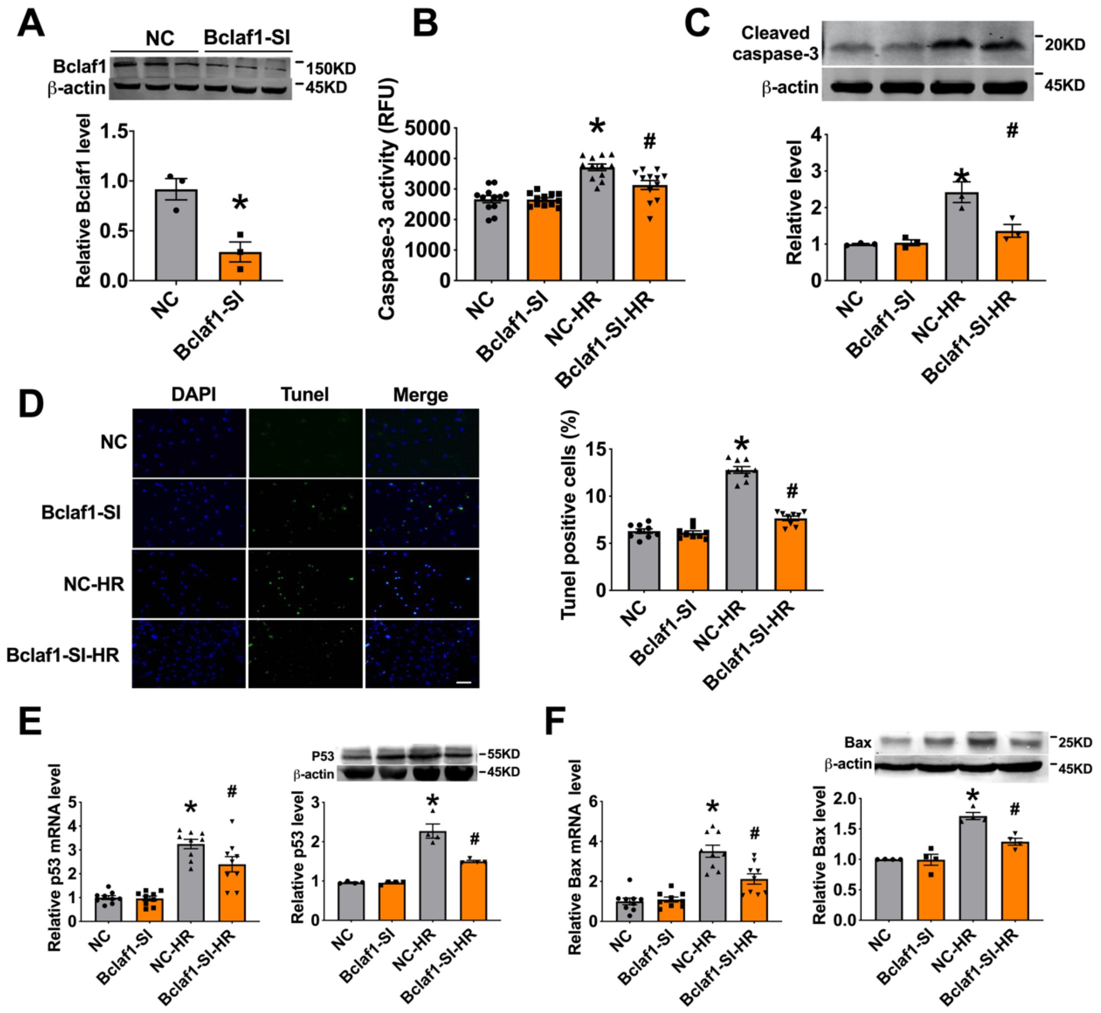

Supplementary Figure 9. Knockdown of Bclaf1 alleviated apoptosis of cardiac myocytes subjected to hypoxia/reoxygenation. (A) Bclaf1 protein level in cardiomyocytes transfected with its siRNA. N=3. *P<0.05 versus NC group. P-values were determined by unpaired t test. **(B, C)** Caspase-3 activity and cleaved caspase-3 protein level. N=12 from 3 independent cultures for caspase-3 activity, and N=3 for cleaved caspase-3 protein level. *P<0.05 versus NC, $^{\#}P$<0.05 versus NC-HR. P-values were determined by one-way ANOVA test followed by Bonferroni post hoc analysis. **(D)** Cardiomyocyte apoptosis by TUNEL assay (scale bar: 20μm). N=10, from 3 independent cultures. *P<0.05 versus NC, $^{\#}P$<0.05 versus NC-HR. P-values were determined by one-way ANOVA test followed by Bonferroni post hoc analysis. **(E, F)** The mRNA and protein levels of p53 and Bax. N=9 for mRNA by qRT-PCR, and N=3 for protein expression by Western blot, from 3 independent cultures.

*$P<0.05$ versus NC, #$P<0.05$ versus NC-HR. P-values were determined by one-way ANOVA test followed by Bonferroni post hoc analysis. Data represent the mean ± SEM, n numbers are given in parentheses.

Supplementary Figure 10

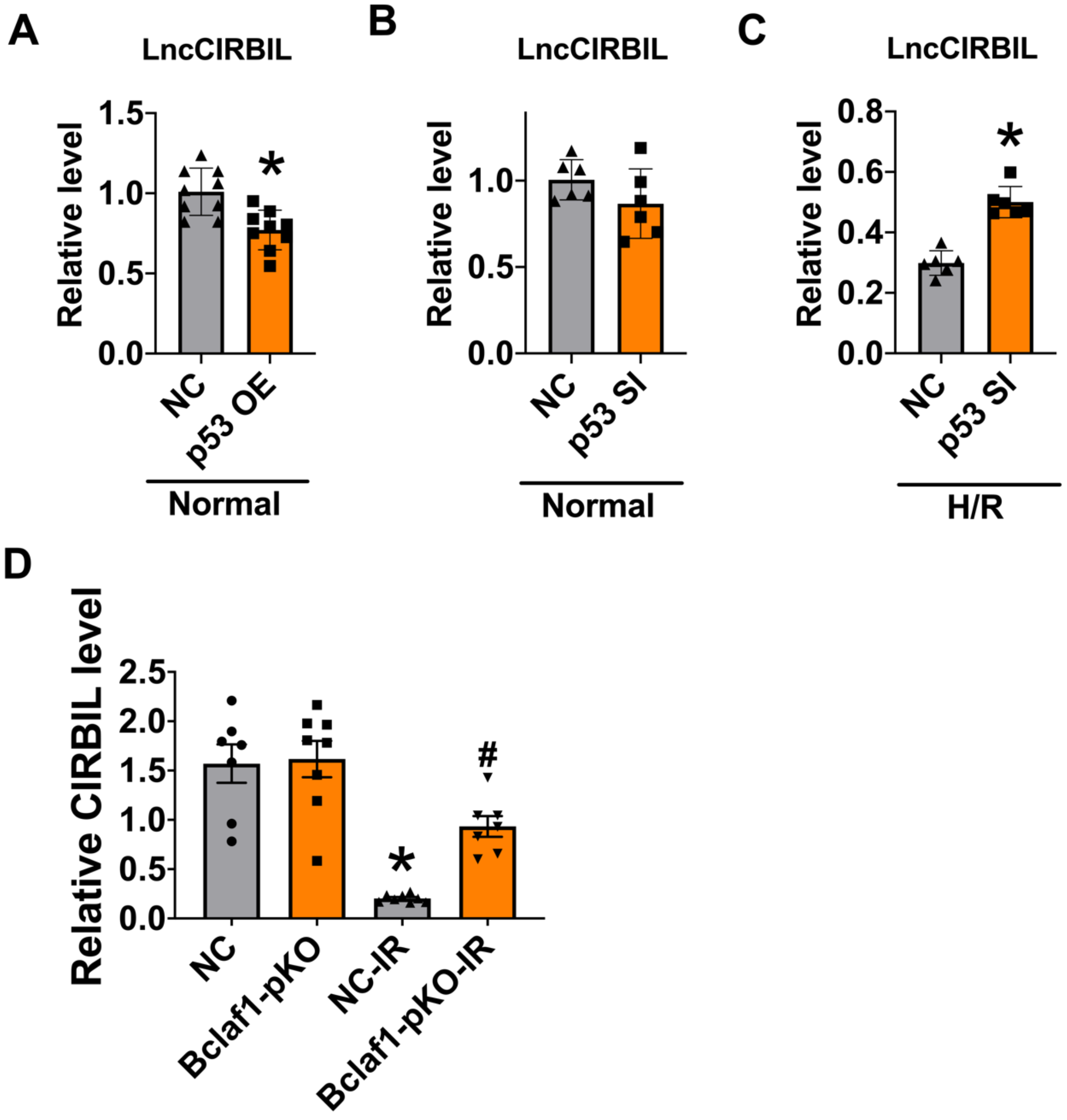

Supplementary Figure 10. p53 regulates the expression of lncCIRBIL. (A) The levels of lncCIRBIL in cardiomyocytes with p53 overexpression. N=9 from 3 independent cultures. *P<0.05 versus negative control (NC, empty plasmid). P-values were determined by unpaired t test. **(B, C)** The levels of lncCIRBIL in cardiomyocytes with p53 knockdown under normal and H/R conditions. N=6 from 3 independent cultures. *P<0.05 versus negative control (NC). **(D)** Effects of bclaf1 partial knockdown on the expression of LncCIRBIL. NC N=7, Bclaf1-pKO N=8, NC-I/R N=8, Bclaf1-pKO-I/R N=7. *P<0.05 versus NC; $^{\#}P$<0.05 versus NC-IR. P-values were determined by one-way ANOVA test followed by Bonferroni post hoc

analysis. Data represent the mean ± SEM, n numbers are given in parentheses.

Supplementary Tables

Supplementary Table 1. Real-Time PCR sequence of primers

Primer name	Primer sequence
Bclaf1(mouse)	Forward: 5’- TTGGATGTGACAACTGCAACG-3’<br>Reward: 5’- GTGGCGGTAACGAATCTCCAG-3’
Bax (mouse)	Forward: 5’- TGGAAGAAGATGGGCTGAGG-3’<br>Reward: 5’- TTCCCACCCCTCCCAATAAT-3’
p53 (mouse)	Forward: 5’-TGGAGGAGTCACAGTCGGAT-3’<br>Reward: 5’- CAGTGAGGTGATGGCAGGAT -3’
LncCIRBIL(mouse)	Forward: 5’-AAGAAGGTGGTGAAGCAGGC-3’<br>Reward: 5’-TCCACCACCCAGTTGCTGTA-3’
LncCIRBIL(human)	Forward: 5’- TAGTGAAGGGGAAAGGTGGC-3’<br>Reward: 5’- AAGCTCCACTCCGTGCTAAT-3’
Actin (mouse)	Forward: 5’-GACAGCAGTTGGTTGGAGCA-3’<br>Reward: 5’-TTGGGAGGGTGAGGGACTTC-3’

Supplementary Table 2. Information of antibodies used in the study

Antibody	Dilution	Company	Catalog Number
Bclaf1	WB 1:1000<br>IF 1:500	Abcam,<br>UK	Ab181240
Bax	WB 1:1000	Proteintech Beijing<br>China	Cat50599-2-lg
β-actin	WB 1:1000	Cell Signaling<br>Technology Boston USA	# 4970
Lamin-b	WB 1:500	Wanleibio Shenyang<br>China	WL01775
p53	WB 1:1000	Cell Signaling<br>Technology Boston USA	# 2524
Tublin	WB 1:1000	Absin<br>China	Abs830032
