# Supplementary material for: The long noncoding RNA lncCIRBIL disrupts the nuclear translocation of Bclaf1 alleviating cardiac ischemia–reperfusion injury
Source: Nat Commun. 2021 Jan 22;12:522. doi: 10.1038/s41467-020-20844-3 (PMC7822959; doi:10.1038/s41467-020-20844-3)
Supplement: Supplementary file 4 — Reporting Summary [file 41467_2020_20844_MOESM4_ESM.pdf]

nature research

Corresponding author(s): Zhenwei Pan, Baofeng Yang

Last updated by author(s): Oct 3, 2020

Reporting Summary

Nature Research wishes to improve the reproducibility of the work that we publish. This form provides structure for consistency and transparency in reporting. For further information on Nature Research policies, see our Editorial Policies and the Editorial Policy Checklist.

Statistics

For all statistical analyses, confirm that the following items are present in the figure legend, table legend, main text, or Methods section.

n/a	Confirmed	
☐	☒	The exact sample size (*n*) for each experimental group/condition, given as a discrete number and unit of measurement
☐	☒	A statement on whether measurements were taken from distinct samples or whether the same sample was measured repeatedly
☐	☒	The statistical test(s) used AND whether they are one- or two-sided<br>*Only common tests should be described solely by name; describe more complex techniques in the Methods section.*
☒	☐	A description of all covariates tested
☒	☐	A description of any assumptions or corrections, such as tests of normality and adjustment for multiple comparisons
☐	☒	A full description of the statistical parameters including central tendency (e.g. means) or other basic estimates (e.g. regression coefficient) AND variation (e.g. standard deviation) or associated estimates of uncertainty (e.g. confidence intervals)
☐	☒	For null hypothesis testing, the test statistic (e.g. *F*, *t*, *r*) with confidence intervals, effect sizes, degrees of freedom and *P* value noted<br>*Give P values as exact values whenever suitable.*
☒	☐	For Bayesian analysis, information on the choice of priors and Markov chain Monte Carlo settings
☒	☐	For hierarchical and complex designs, identification of the appropriate level for tests and full reporting of outcomes
☒	☐	Estimates of effect sizes (e.g. Cohen's *d*, Pearson's *r*), indicating how they were calculated

Our web collection on statistics for biologists contains articles on many of the points above.

Software and code

Policy information about availability of computer code

Data collection	NO software was used
Data analysis	Graphpad Prism 8.0, Image pro plus 6.0.

For manuscripts utilizing custom algorithms or software that are central to the research but not yet described in published literature, software must be made available to editors and reviewers. We strongly encourage code deposition in a community repository (e.g. GitHub). See the Nature Research guidelines for submitting code & software for further information.

Data

Policy information about availability of data

All manuscripts must include a data availability statement. This statement should provide the following information, where applicable:

- Accession codes, unique identifiers, or web links for publicly available datasets
- A list of figures that have associated raw data
- A description of any restrictions on data availability

The data that support the findings of this study and unique materials are available from the corresponding authors upon reasonable request.

Field-specific reporting

Life sciences study design

All studies must disclose on these points even when the disclosure is negative.

Sample size	No statistical test was used to predetermine sample size. Sample size was determined based on the experimental results that we obtained from preliminary experiments. In vivo studies, we used standard sample sizes reported in the literature previously in mouse studies. The numbers of performed experiments were indicated in each figure legend. The sample size for whole animal experiments was set to be >4 mice for each group, and for molecular biology experiments, the sample size for set to 3-6 for each group.
Data exclusions	The data from the animals died before the completion of the whole experimental procedures were excluded from our data analysis.
Replication	For cellular and molecular experiments, each single measurement was performed in triplicate and the results were consistently reproducible
Randomization	All animals and cells were randomly assigned to experimental groups.
Blinding	The experimental designers and experimenters/data analysts were double blinded.

Reporting for specific materials, systems and methods

We require information from authors about some types of materials, experimental systems and methods used in many studies. Here, indicate whether each material, system or method listed is relevant to your study. If you are not sure if a list item applies to your research, read the appropriate section before selecting a response.

Materials & experimental systems

n/a	Involved in the study	
☐	☒	Antibodies
☒	☐	Eukaryotic cell lines
☒	☐	Palaeontology and archaeology
☐	☒	Animals and other organisms
☐	☒	Human research participants
☒	☐	Clinical data
☒	☐	Dual use research of concern

Methods

n/a	Involved in the study	
☒	☐	ChIP-seq
☒	☐	Flow cytometry
☒	☐	MRI-based neuroimaging

Antibodies

Antibodies used	Bclaf1 (EPR9980, Abcam, Cambs, UK), cleaved-Casepase-3 (#9664, CST, Boston, USA), Bax (50599-2-lg, Proteintech, Beijing, China),p53 (#2524,CST, Boston, USA), β-actin (# 4970, CST, Boston, USA), Lamin-b(A1910, abclonal, Wuhan, China), tubulin (abs830032, absin, Shanghai, China), IRDye® 800CW Goat anti-Rabbit IgG Secondary Antibody (AB_2651127,Lincoln, NE, USA), IRDye® 800CW Goat anti-Mouse IgG Secondary Antibody (AB_2687825, Lincoln, NE, USA), Alexa Fluor® 488 (# A-11034, invitrogen,California, USA)
Validation	All the primary antibody for the species and application statement on the manufacturer's websites. Bclaf1 see https://www.abcam.cn/btf-antibody-epr99802-ab181240.html, Cleaved-Casepase-3 see https://www.cellsignal.cn/products/primary-antibodies/cleaved-caspase-3-asp175-5a1e-rabbit-mab/9664?N=4294956287&Ntt=%239664&fromPage=plp, Bax see https://www.ptgcn.com/Products/BAX-Antibody-50599-2-Ig.htm, P53 see https://www.cellsignal.cn/products/primary-antibodies/p53-1c12-mouse-mab/2524?N=4294956287&Ntt=%232524&fromPage=plp, β-actin see https://www.cellsignal.cn/products/primary-antibodies/b-actin-13e5-rabbit-mab/4970?N=4294956287&Ntt=%23+4970%2C&fromPage=plp, Lamin-b see https://abclonal.com.cn/catalog/A1910, tubulin see https://www.absin.cn/anti-tubulin-monoclonal-antibody/abs830032.html, Goat anti-Rabbit IgG Secondary Antibody see https://www.licor.com/bio/reagents/irdye-800cw-goat-anti-rabbit-igg-secondary-antibody,Goat anti-Mouse IgG Secondary Antibody see https://www.licor.com/bio/reagents/irdye-800cw-goat-anti-mouse-igg-secondary-antibody, Alexa Fluor® 488 see https://www.thermofisher.com/order/genome-database/dataSheetPdf?producttype=antibody&productsubtype=antibody_secondary&productId=A-11034&version=123

Animals and other organisms

Policy information about studies involving animals; ARRIVE guidelines recommended for reporting animal research

Laboratory animals	Male C57BL/6 mice (8 weeks of age) weighing 25 g were obtained from Beijing Vital River Laboratory Animal Technology Co., Ltd. (Beijing, China). LncRNA-CIRBIL cardiac-specific transgenic over-expression, global knockout mice and cardiac-specific Bclaf1 over-expression mice were generated by Cyagen Biosciences Inc (Guangzhou, China).All animals were maintained in accordance with guidelines: Standard chow diet and water were offered ad libitum; for housing sterilized plastic cages under specific pathogen-free conditions were used; as housing conditions 23 ± 3 °C, 12/12 light/dark cycle, 30-70% humidity and <400 lux was maintained.
Wild animals	The study does not involve any wild animals

Field-collected samples	The study does not involve any samples collected from Field.
Ethics oversight	The experimental protocols involving the use of animals in this study were approved by the Animal Care and Use Committee of Harbin Medical University (HMUIRB3005719)

Note that full information on the approval of the study protocol must also be provided in the manuscript.

Human research participants

Policy information about studies involving human research participants

Population characteristics	In the First Affiliated Hospital of Harbin Medical University (Harbin, China), whole blood (WB) samples (1 mL per patient) were drawn from the study subjects via a direct venous puncture into tubes containing sodium citrate. For AMI, peripheral blood samples were collected within an average ischemic time of 3.5 h prior to blood draw. The samples were used directly for RNA isolation.
Recruitment	Between June 2020 and September 2020 32 AMI patients and 32 non-AMI control subjects presented to the First Affiliated Hospital of Harbin Medical University (Harbin, China). AMI was diagnosed based on combination of several parameters: ischemic symptoms plus increased cardiac troponin I (cTnI) and creatine kinase-MB (CKMB), appearance of pathological Q wave, and ST-segment elevation or depression defined by the European Society of Cardiology/American College of Cardiology. Informed written consents were obtained from all participants and all investigations conformed to the principles of the Declaration of Helsinki. The study protocols were procured in accordance with the guidelines of and approved by the Ethics Committee of the Harbin Medical University.
Ethics oversight	The experimental protocols involving the use of human blood sample in this study were approved by the Ethics review Committee of Harbin Medical University (HMUIRB3005719)

Note that full information on the approval of the study protocol must also be provided in the manuscript.
